# Community Hospice Nurses’ Perspectives on Needs, Preferences, and Challenges Related to Caring for Children With Serious Illness

**DOI:** 10.1001/jamanetworkopen.2021.27457

**Published:** 2021-10-04

**Authors:** Amy S. Porter, Kristina Zalud, Jacob Applegarth, Cameka Woods, Melanie Gattas, Emily Rutt, Karen Williams, Justin N. Baker, Erica C. Kaye

**Affiliations:** 1 Division of Quality of Life and Palliative Care, Department of Oncology, St Jude Children’s Research Hospital, Memphis, Tennessee; 2 St Louis Children’s Hospital, Washington University School of Medicine, St Louis, Missouri; 3 Indiana University School of Medicine, Indianapolis

## Abstract

**Importance:**

Many of the 50 000 children who die annually are eligible for provision of community-based hospice care, yet few hospice organizations offer formal pediatric services. Population-level data demonstrate that hospice nurses lack training, experience, and comfort in caring for children, but their specific educational needs and preferences are poorly understood.

**Objective:**

To assess the pediatric-specific training and support needs of hospice nurses caring for children in the community.

**Design, Setting, and Participants:**

For this qualitative study, 41 nurses were purposively seletected from a population-level cohort of 551 hospice nurses who completed a previous mixed-methods survey; these 41 nurses participated in semistructured interviews between February and April 2019. Hospice nurses were recruited from all accredited hospice organizations in Tennessee, Mississippi, and Arkansas that offer care to pediatric patients. Content analysis of interview transcripts was conducted.

**Main Outcomes and Measures:**

The interview guide probed for topics related to prior pediatric hospice training experiences, desires and preferences for training, and perceived barriers to training.

**Results:**

Interviews were conducted with 41 nurses representing different geographic regions and levels of comfort with pediatric hospice provision (as self-reported in the prior survey). Thirty-eight of the nurses were women (92.7%), with a median age of 40-49 years (range, 20-29 to ≥60 years) and median tenure of 5-9 years (range, <1 to ≥20 years) as a hospice nurse. Respondents included 1 American Indian or Alaska Native nurse (2.4%), 1 Black nurse (2.4%), and 39 White nurses (95.1%). Across interviews, most hospice nurses reported feeling uncomfortable caring for children with serious illness, and all nurses used language to express the immediacy behind the need for pediatric-specific training and support. Nurses explained why further training and support are needed and clear preferences for who should provide the education, educational modalities, and specific training topics. Nurses also articulated barriers to training and support opportunities and proposed innovative suggestions for overcoming these challenges. Notably, nurses emphasized the need for connection with experts, a sense of community, and solidarity to support frontline clinicians providing end-of-life care to children in the community.

**Conclusions and Relevance:**

In this qualitative study, community hospice nurses expressed an urgent need for improvements in pediatric-specific training opportunities and support, clear preferences for how education should be provided, and recommendations for circumventing barriers to training. These findings are a call to action for the palliative care community to collaborate in rapid implementation of educational programs and networks to systematically support hospice nurses caring for children in the community.

## Key Points

**Question:**

What are the needs and preferences of community hospice nurses for pediatric training and support?

**Findings:**

In this qualitative study, semistructured interviews with a purposive sample of 41 community hospice nurses revealed that most hospice nurses are uncomfortable caring for children with serious illness and perceive their insufficient pediatric training and support as targetable gaps necessitating urgent action. Nurses also offered recommendations for training and support in pediatric care provision and identified significant barriers to such training.

**Meaning:**

In this study, community hospice nurses expressed an urgent need for accessible pediatric-specific education and training opportunities.

## Teaser

This qualitative study assesses the opinions of community hospice nurses regarding the provision of hospice care for children with serious illness.

## Introduction

Approximately 500 000 children in the US have a serious illness, of whom 10% die annually,^1^ and of those, 11% die at home^2^—a number that continues to increase.^3^ Optimal provision of home-based hospice services can lessen symptom burden and improve quality of life,^4^ reduce parental psychosocial stress,^5,6^ decrease costs,^7,8^ and limit clinician distress.^6^ Accordingly, the Institute of Medicine and the American Academy of Pediatrics advocate for the early integration of palliative and hospice services for children with serious illness.^9,10,11^

Presently, however, only 1 in 10 dying children receive hospice care, usually through adult organizations.^9,10,11^ Although National Hospice and Palliative Care Organization surveys have shown that more than three-quarters of adult hospices serve pediatric patients, only 14% have pediatric-specific programs.^12^ Johnston et al^13^ revealed that bereaved parents desired home death but reported that lack of home support made dying at home challenging, with poor symptom management leading to returns to the hospital. Caring for children at the end of life poses unique challenges that demand pediatric-specific knowledge. Alongside balancing symptom management, communication, and care coordination, hospice clinicians also carry an emotional burden, which may contribute to stress and moral distress.^14,15^

Unfortunately, few community hospice nurses receive pediatric-specific training, resources, and support.^16,17,18^ A previous study^16^ surveyed more than 550 hospice nurses across 71 hospice agencies that offer care to children in the tristate region of Tennessee, Arkansas, and Mississippi and found that nearly 90% of nurses had no training in pediatric palliative or hospice care; 50% had no pediatric hospice experience; those with exposure to pediatric hospice care described limited training (eg, a 2-day course); and few had opportunities to maintain or build their skill sets. Unsurprisingly, hospice nurses reported overwhelming discomfort with pediatric-specific care^16,17,18^ and a strong desire for further education, resources, and support specific to the care of pediatric patients. In fact, many nurses requested training on any or all topics.^18^ Qualitative analysis of short-answer survey items identified several key gaps in knowledge self-reported by hospice nurses, including pediatric medication use and dosing, physical symptom assessment and management, psychosocial assessment and management, and communication.^18^ Nonetheless, hospice nurses’ educational needs and preferences regarding pediatric-specific training, resources, and support systems are not well understood.

To address this deficit, an interdisciplinary collaborative of pediatric palliative care and hospice physicians, advanced practice health care professionals, and nurses partnered with researchers to develop a qualitative study of hospice nurses’ pediatric-specific training needs and preferences. The ultimate goal of the study was to present nurse-driven recommendations for optimizing pediatric educational resources, training programs, hospice policies, and supportive interventions to improve the overall provision of community-based hospice care to children with serious illness and their families.

## Methods

This qualitative study was reviewed and approved by the institutional review board at St Jude Children’s Research Hospital, and all participants provided formal verbal consent. This study followed the Consolidated Criteria for Reporting Qualitative Research (COREQ) reporting guideline.

### Preceding Survey Study

Methods for the 2018 population-level survey study of hospice nurse experiences and comfort with pediatric hospice provision have been previously described.^16,17,18^ Briefly, we identified all accredited hospice organizations that offer services to pediatric patients in the tristate region of Tennessee, Mississippi, and Arkansas, comprising an institutional catchment area with notably poor access to hospice organizations, with approximately one-quarter to one-third of individuals lacking services within a 30-minute drive.^19^

### Interview Guide Development and Content

Following review of the literature related to barriers to provision of pediatric hospice care to children in the community,^20,21,22,23,24,25,26,27,28,29^ a semistructured interview guide was drafted by a multisite, interdisciplinary team of pediatric palliative care and hospice clinicians and researchers. Interview questions were iteratively reviewed and refined by a panel of stakeholders, including 2 pediatric hospice physicians and 5 pediatric hospice nurses, with serial item assessment for content and construct validity until consensus was achieved.^30^ The final interview guide (eFigure in the Supplement) included questions to assess comfort levels with, training and experience in, and barriers to caring for children with serious illness. Open-ended questions were designed to explore hospice nurses’ training and support needs and preferences in pediatric palliative and hospice care.

### Participant Selection, Consent, and Interview Processes

Prior to this interview study, a mixed-methods survey was distributed to 551 community hospice nurses from 71 hospice agencies across the region, 226 of whom indicated willingness to have a follow-up interview. Survey participants self-identified their race and ethnicity, selecting from a provided list of categories. Purposive sampling^31^ was used to select a cohort of 41 nurses representing different self-reported levels of comfort with pediatric hospice care provision, self-identified as very uncomfortable, somewhat uncomfortable, somewhat comfortable, and very comfortable. Nurses were randomly selected until more than 10 were enrolled from each stratum. Only 3 nurses declined interviews. Email invitations were sent to selected nurses, and for those who wished to participate, an audio-recorded telephone interview was conducted by a female physician (A.S.P.) with expertise in pediatric complex care and a doctoral degree in anthropology, with no others present on the telephone call. Before beginning each interview, the interviewer introduced herself and her background and obtained formal verbal consent. Semistructured interviews were conducted over a period of 2 months between February and April 2019. Interviews were transcribed verbatim by trained medical transcriptionists.

### Codebook Development, Coding, Adjudication, and Analysis

Content analyses were conducted using MAXQDA (VERBI Software) to organize data.^32^ The eTable in the Supplement presents each step of codebook development, piloting, coding, and synthesis and validation, all of which were performed in accordance with COREQ guidelines.^33^ Interviews continued until data saturation was reached.

## Results

A total of 41 community hospice nurses completed interviews. Table 1 presents self-reported participant demographic data and clinical practice variables. Thirty-eight of the nurses were women (92.7%) and 3 were men (7.3%), with a median age of 40-49 years (range, 20-29 to ≥60 years) and median tenure of 5-9 years (range, <1 to ≥20 years) practicing as a hospice nurse. Respondents included 1 American Indian or Alaska Native nurse (2.4%), 1 Black nurse (2.4%), and 39 White nurses (95.1%). Interview duration ranged from 20 to 60 minutes.

**Table 1.** Self-reported Participant Demographic Characteristics and Clinical Practice Variables Among 41 Participants

Survey item or response	No. (%)
Sex	
Female	38 (92.7)
Male	3 (7.3)
Race	
American Indian or Alaska Native	1 (2.4)
Arabic or Middle Eastern	0
Asian or Pacific Islander	0
Black	1 (2.4)
White	39 (95.1)
Other	0
Ethnicity	
Hispanic	0
Non-Hispanic	41 (100)
Age, y	
≤19	0
20-29	5 (12.2)
30-39	10 (24.4)
40-49	10 (24.4)
50-59	12 (29.3)
≥60	4 (9.8)
State	
Arkansas	12 (29.3)
Mississippi	10 (24.4)
Tennessee	19 (46.3)
Time as a nurse, y	
<1	0
1-4	5 (12.2)
5-9	9 (22.0)
10-19	9 (22.0)
≥20	18 (43.9)
Time as a hospice nurse, y	
<1	7 (17.1)
1-4	12 (29.3)
5-9	11 (26.8)
10-19	10 (24.4)
≥20	1 (2.4)
Comfort level	
Very uncomfortable	7 (17.1)
Somewhat uncomfortable	13 (31.7)
Somewhat comfortable	12 (29.3)
Very comfortable	9 (22.0)

### “I Don’t Ever Really Feel Comfortable”: Hospice Nurse Discomfort With and Lack of Training in Provision of Pediatric Care

Many nurses reported that they almost always feel uncomfortable with or underprepared to care for children. When asked, “Will you please think back to the last time you were working with a child with palliative or hospice care needs in which you felt uncomfortable or underprepared and tell me about that,” one interviewee responded: “About every day,” and another explained: “I don’t ever really feel comfortable.” One nurse described the apprehension that she and her colleagues feel: “We all get kind of a pit in our stomach of ‘How am I going to take care of this child and their family?’” Another nurse offered: “I have such a lack of education and knowledge in taking care of children in hospice that if I got them as a patient, I would have no idea.” Reflecting on a particular experience caring for a pediatric patient, one nurse shared: “This day—it was a learning curve for everybody, and I don't feel like we had the training, the tools, or the resources to benefit the patient.” These feelings of discomfort and lack of preparation were reported more frequently by nurses who, in the preceding survey, had self-identified as either somewhat or very uncomfortable compared with those who had self-identified as either somewhat or very comfortable. Importantly, many conveyed that this discomfort makes nurses unwilling to care for a child at the end of life.

Table 2 presents specific facets of pediatric care with which hospice nurses reported feeling uncomfortable. Themes included communicating with a sick child and the child’s parents, communicating with siblings, caring for pediatric patients without sufficient pediatric physician support, symptom management in pediatric patients (as compared with adult patients), anticipating what is normal for pediatric patients overall and especially at the end of life, and preparing for and witnessing a child’s death.

**Table 2.** Elements of Pediatric Care Provision With Which Hospice Nurses Report Feeling Uncomfortable and Rationale for Further Training in Pediatric Hospice and Palliative Care

Theme	Supporting quotations
**Elements of pediatric care provision with which hospice nurses report feeling uncomfortable**	
Communication with a sick child and parents	“I feel more uncomfortable working with those patients…even how to communicate with them. What to say to the patient…or the parent because that is a whole different element in that kind of care. So, to communicate and medically handle the child is just a different—I feel like I’m a lot more hands-off than I would be with my adult patients because it’s like, ‘Oh, I don’t feel comfortable with this.’ If it’s an infant, the parent would probably hold the infant more than I would ever feel comfortable. Or if it’s a younger child, I would probably end up directing my conversation to the parent [more] than involving the child, which I feel like is due to my comfort level rather than how it should be.” “I think the biggest thing in pediatrics is we forget about the kids…, how intuitive they are. They know they’re dying; it’s not a secret. And the parents may want it to be a secret, but the children know. And most of the time when the parents are out of the room the kid will let you know they know. I think it’s so important that you acknowledge the parents but more importantly acknowledge the patient, the child. And so many nurses forget. I forget.”
Communication with siblings	“I have a 15-year-old that I have to take care of. There’s 4 brothers in the home and they’re all different ages, and it’s hard to approach the [siblings] because I’m not really sure how to approach, how…to teach them about life and death.”
Caring for pediatric patients without sufficient pediatric physician backup	“I don’t know that we would accept pediatric patients… I would have a very difficult time saying, ‘Yes, we will accept this pediatric patient,’ if I didn't know we didn’t have a strong pediatric physician providing the clinical resources we need… The clinical background and making sure we’re dosing correctly, we’re recognizing signs and symptoms, we’re managing them to the best of our ability. In the home, to keep the patient comfortable is important.”
Symptom management in pediatric patients (as compared with adult patients)	“I felt just so helpless. This poor little baby was like 5 days old. He was just kind of gasping for air and I felt like he was drowning in his secretions. I just didn’t have all the stuff there that I needed. I kept thinking he was going to pass anytime and it just kept going on for hours and hours. So, I said at that time I’m never going to take care of a baby again. I do admissions now. I’m going to get them what they need up front just in case.” “[Pediatric care] is so different from adults. I know symptom management–wise, immediately pretty much what I need to do for an adult. With kids, I’m double-checking myself. The confidence is not there, especially with symptom management.”
Anticipating the “norm” for pediatric patients overall and at the end of life	“[I] think honestly, for me, the hospice piece, the death piece—I’m very comfortable with that, but where I’m uncomfortable, is pediatrics in general. Kids, I just don’t know about kids. Growth and development, where they should be. Really my only pediatric experiences are when I was raising my kids. So, it’s really just more of not knowing kids really. What’s the normal respiratory rate for a child, what’s the normal blood pressure, what’s the normal heart rate? Even those really basic things, that [with] no pediatric experience, I don’t really know what a baseline should be.” “I had an 8-year-old and was always very unsure of myself as far as, ‘Am I assessing the patient correctly? What are the normal vital signs for a pediatric patient vs somebody that—my typical geriatric population?... When this child entered the dying process, is this, does this look the same as it does for an adult?’”
Preparing for and witnessing a child die	“Having a conversation and telling someone that their child is dying is a very uncomfortable feeling. No matter how many times I’ve had that conversation with adult or geriatric patients, it’s very different when it’s a child. To [ensure] they were making the right decisions on behalf of the child without inflecting what my thoughts and feelings were, knowing the clinical picture probably clearer than they did, was very difficult. That was very uncomfortable to me because it was not about me telling them what I would do. It was about them coming to the right decision for them… I don’t know that I have that training and background just to help someone walk through that.” “I don’t even know what to do, and nothing naturally came to me, [such] as how I should handle it. Should I offer to redress the baby? Things like that, just… Even postmortem care felt very uncomfortable because it was just so foreign to me with no education.”
**Rationale for further training in pediatric palliative and hospice care**	
Need to absorb increasing pediatric hospice referrals	“They built a children’s hospital up here, a branch of [a larger children’s hospital]. So, we are starting to see a lot more pediatric patients. I think when you all’s survey came out, the initial one, I was like, ‘Oh my gosh. This is so needed now more than any other time,’ because in the past, people would be [geographically distant] and come all the way [here, across state boundaries]. So, in our little area, we are starting to see more pediatric patients because there’s this hospital here now and they’re referring to us more.” “They know at [the children’s hospital] that [our agency] will take a pediatric patient, so it seems like we get a lot of referrals from them.”
Geographic isolation of many hospice nurses from clinicians trained in pediatric palliative and hospice care	“Sometimes we feel like we’re inventing it out here in Tennessee and southwest Virginia.”
Difficulty of finding hospices willing to accept pediatric patients	“I truly don’t know of a hospice company in our area that really takes care of children.” “I think it’s getting harder and harder to find hospices that’re really willing to take care of children. In the way that I need to be taken care of.”
Given the relative rarity of pediatric patients as compared to adults, dearth of opportunities for gaining experience and thus building skills and confidence	“I think that’s because our pediatric volume is so low, every time that we do get a pediatric referral, it’s a very uncomfortable thing because we don’t do it a whole lot.” “It’s hard when there is not that many in the area... Like I said, I’ve had 3 or 4 patients in 4 years. It’s just hard to get that comfort level.”
Fundamental difference between caring for children and caring for adults	“Pediatrics is a bitch. And if you don’t feel comfortable around that and never really dealt with kids, then you do need, in my opinion, you need more training and more knowledge and more not only the book knowledge, but that hands-on…”

### “We Feel Like We’re Inventing It Out Here”: Rationale for Further Training in Pediatric Palliative and Hospice Care

Universally, nurses reported that their training in pediatric palliative and hospice care had been limited. One nurse shared: “I mean, I got a whole week and a half of training total in hospice, and then I had to wing it on my own.” Nurses explained why further pediatric training is essential and urgent, including 5 key themes (Table 2): the need to absorb increasing pediatric hospice referrals, the geographic isolation of many hospice nurses from clinicians trained in pediatric care, the difficulty of finding hospices willing to accept pediatric patients, the dearth of opportunities for gaining experience and building skills and confidence given the relative rarity of pediatric patients as compared with adults, and the fundamental difference between caring for children and caring for adults. One nurse explained: “Children aren’t little adults. There’s comparison of a child to an adult, and you have to approach them different[ly].” Two nurses emphasized that acceptance of concurrent care is one key way pediatric palliative and hospice care differs from that of adults and recommended as a topic for pediatric-specific curricula: “Something that is a gray area for me is current care. There was always that question—because in the adult world, there’s a line. There’s definitely a line of what you can’t do and what you can. Peds is a whole different world… What is acceptable under hospice? How far can we go? I mean, that’s my understanding [that] anyone can continue to get as much aggressive treatment as they need or as they want….”

### “So I’m Not in This Alone”: Preferences for Educators and Access to Expertise

Nurses described specific preferences for educators (Table 3). Many emphasized the importance of incorporating experienced pediatric clinicians as teachers: “I would want a pediatric palliative care physician or nurse practitioner to come in and talk to us specifically about the different things regarding pediatric patients. And really share those experiences and tell us, kind of help us walk through what would work and what wouldn’t work and just different options.” One nurse stated that her agency would not accept pediatric patients if they did not have pediatric palliative care physician support: “I would have a very difficult time saying, ‘Yes, we will accept this pediatric patient,’ if I didn’t know we didn’t have a strong pediatric physician providing the clinical resources we need… [We need support in terms of] clinical background and making sure we’re dosing correctly, [that] we’re recognizing signs and symptoms, [that] we’re managing them to the best of our ability.” Many nurses also underscored the importance of multidisciplinary pediatric palliative care clinician support for real-time learning: “It’s just being available because we kind of feel like we’re in the dark sometimes, and we don’t really know where we’re supposed to be turning and what we’re supposed to be doing. And it’s very helpful to have that direct line that you can call and just say, hey look, I don’t know what’s going on here.” Many requested access to more experienced nurses to provide them with hands-on training.

**Table 3.** Nurse Preferences for Training in Pediatric Palliative and Hospice Care

Facet of training	Preferences
Teachers	Pediatric palliative care experts (eg, experienced hospice nurses, nurse educators, nurse practitioners, and physicians) Hospice nurses with pediatric experience as “super trainers” Parents of seriously ill children
Approach or modality	In-person and face-to-face learning and didactics (eg, lectures, seminars, workshops, training courses, conferences, or small group discussions) Establishing a learning community: value of bringing together like-minded clinicians to learn together in solidarity Experience-based education: recognizing that past experiences caring for children with serious illness is a good foundation for PPHC learning Ease: ready access to tools and resources Credit: option to receive certification or formalized credit for education
Topics	Assessing and responding to common symptoms experienced by children in hospice Cultural competency (eg, caring for patients and families of different races, ethnicities, religions, sexual orientations, or gender identifications) Navigating difficult conversations (eg, how to talk to children about their illness; how to talk with families; how to talk about end of life with children and families; goals of care; decision-making; or patient and family empowerment) Caring for and supporting the whole family unit, as well as the dying child Provision of grief and bereavement services How to engage in an age-appropriate way (eg, conducting a visit in the context of the child’s developmental stage; providing care in a way that is not scary for a pediatric patient) Pediatric pathophysiology and common diagnoses Provision of nonpharmacologic approaches to manage symptoms (eg, any strategy to provide comfort apart from administration of a medication; complementary or alternative therapies; or psychosocial support) Strategies for taking care of oneself (eg, preventing burnout; developing resilience; or establishing boundaries) Familiarity with pediatric devices and equipment (eg, pumps; PEG tubes; ventilators; or tracheostomies) Common issues at the end of life in children (eg, how disease progression and end of life differ in children vs adults) Balancing authentic emotions and professionalism (eg, “it’s ok to cry”; “it’s ok to be human”; or “it’s ok to be apprehensive”) Provision of end-of-life or comfort care, including facilitation of death with dignity and provision of postmortem care Provision of legacy-building activities and support (eg, activities or conversations designed to help family members remember the child after death)

Abbreviations: PEG, percutaneous endoscopic gastrostomy; PPHC, pediatric palliative and hospice care.

Last, some nurses described parents of seriously ill children as fundamental teachers of best practices in palliative and hospice care. One nurse shared: “Of course, the parents had been trained backwards and forwards, up and down… so, if there was something that I might not have known… As a nurse, you always want to feel like that you know what you are doing when you go out there, but sometimes you don’t and have to rely on that family.”

### “I’m More of a Hands-On, Interactive Learner”: Preferred Learning Modalities and Training Topics

Preferred training modalities are also described in Table 3. In-person learning from a teacher through face-to-face interactions was the most frequent recommendation. Many also suggested establishing a learning community to bring like-minded clinicians together to learn in solidarity. Many mentioned the importance of experiential learning from a teacher with rich past experiences caring for seriously ill children.

Several nurses suggested preceptorships and one-on-one mentoring in the field, in which “somebody goes out with us and watches us do a visit, and then we go back and sit down and talk about [it], not ‘this is what you’re doing wrong’ but ‘hey, this is what you could be doing better.’” Others described creation of a nurse educator role, someone with extensive experience in pediatric palliative and hospice care available at all times for consultation. We imagine a system in which hospice nurses with deep and broad experiences caring for children are paired with hospice nurses with little experience and comfort with pediatric care both to care for patients side-by-side in the field and to develop bidirectional, longitudinal mentor-mentee communication that can empower the less experienced nurse to take on more pediatric patients.

Nurses also identified specific educational topics as crucial to training nurses to provide pediatric hospice and palliative care. Most commonly requested topics fell into 3 categories: technical skills (eg, pediatric pathophysiology, symptom management, pediatric devices and equipment, and common issues for children at the end of life), communication (eg, engaging in age-appropriate ways, caring for the family unit, navigating difficult conversations, providing end-of-life and comfort care, and contextual sensitivity), and resilience (eg, strategies for self-care and boundaries). The Figure presents a synopsis of preferred topics.

**Figure.**
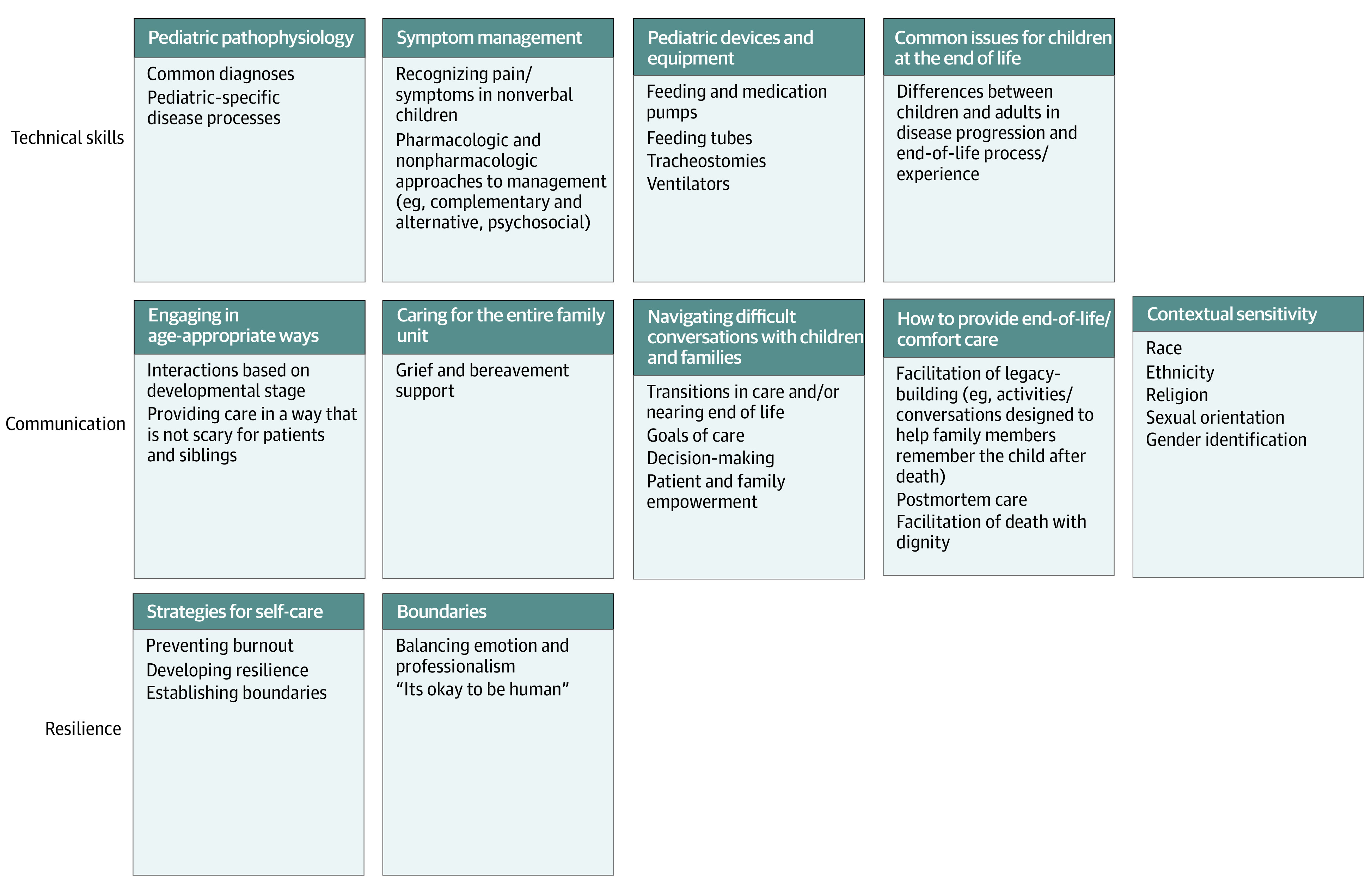
Preferred Training Topics

### “Time Is the Biggest Thing”: Barriers to Training and Solutions for Overcoming Challenges

Nurses identified specific barriers to receiving ideal training (Table 4): lack of time away from professional or personal responsibilities, difficulties subsidizing training costs, lack of awareness of available training opportunities, geographic distance from training opportunities, lack of easy access to centralized resources, perception that hospice agencies do not value such training, and emotional burnout resulting in staff attrition. With foresight and vision, nurses readily suggested strategies to overcome these barriers (Table 4), including staffing and scheduling approaches, funding resources, visibility and accessibility tools, informing hospice agencies at large about how critical these resources are, and resilience training. Many nurses were hopeful; one shared past success in motivating for pediatric-specific training: “I know when I presented it to the last organization that I worked with…when [nurses] really pushed for it and produced sufficient evidence, [the agency] allowed it to happen.”

**Table 4.** Barriers to Hospice Nurse Training in Pediatric Care and Strategies for Overcoming Such Barriers

Barrier	Supporting quotations	Strategies for overcoming barriers
Time: lack of time to participate in educational activities (eg, heavy caseload; overtime work; need to know in advance for adequate staffing; professional responsibilities; or personal obligations)	“Time, just time. Time in general because we are hospice providers. It is always about production, production, production. You’ve got to get up there and see this patient, you got to do this, you got to do all of this. And really being able to have that. Time to sit down for someone to train you in this, to me would be the biggest savior.”	Scheduling: finding convenient times for educational opportunities; planning ahead; allowing for adequate staffing to enable clinicians to attend
Money: financial issues that preclude access to training opportunities (eg, inability to subsidize costs of training)	“Money of course is always an issue. We're a nonprofit so to pay for folks to get an education when we have a fairly high turnover rate is difficult so somethings you know those things are always difficult.” “Our hospital system had begun to offer more education, and then all of a sudden, cuts came through, and they took it all away.”	Funding: providing financial support to attend training, making training more affordable or more cost-effective
Awareness of opportunities: lack of knowledge about educational opportunities (ie, suboptimal advertising; reliance on word of mouth)	“I feel like my biggest barrier is…knowing about them, advertisement of the resources that are out there and the educational opportunities.”	Optimizing accessibility and visibility: making PPHC education content more accessible, available, or visible; creating awareness that training exists and making it convenient for companies to use (online formats, usage of current company infrastructure to disseminate training information)
Geography: location of (and geographic distance from) training opportunities	“I live in a rural area. So, there's really not a lot of opportunity here for that for learning or for those types of hands-on experiences. There's definitely no teaching hospitals or—in this area, other than driving at least an hour and a half or longer.”	
Access: lack of centralized, readily accessible resources	“I had to spend a lot of time trying to figure out how to take the course or how to even find the information on how to take the course, or how to find the literature for the course. It was very, very difficult. I ended up with time and stuff like that and starting to work a lot more, that’s whenever I first started working with the company. I kind of, just gave up on it there, for a little while. I was like, ‘I’ll just read this and this,’ because it was so difficult to find all the pieces that I needed for the course.”	
Value by hospice: perception that hospice organization does not recognize value of PPHC education/training (eg, need for “corporate approval”; funding for travel; or time off for in-service training or external training)	“I think that when it comes down to it, when you’re ready to have the true conversation, ‘Okay, this is going to happen in our agency,’ it causes a real defined line. There’s one side that doesn’t want to have anything to do with pediatric patients.”	Making a big picture argument: convincing leadership of the value added by PPHC training
Burnout: emotional barriers to training (eg, burnout; staff attrition/turnover)	“Building this into a curriculum that an agency buys into, so that they understand the value that it adds. Sometimes it's expected that you learn on your own but not necessarily that they provide that learning for you.”	Optimizing relevance: topics covered during training should be relevant, pragmatic, applicable to daily practice

Abbreviation: PPHC, pediatric palliative and hospice care.

## Discussion

In this qualitative study, nurses providing hospice and palliative care to patients and families across Tennessee, Mississippi, and Arkansas expressed their lack of comfort and training in pediatric care provision, their strong desire for pediatric-specific education, and their belief that there is an urgent need for development of resources and training to improve pediatric palliative and hospice care practice in the community. Additionally, nurses stated clear preferences with respect to source, delivery, and topical content; recognized practical threats to educational resources and programs; and proposed solutions for circumventing or overcoming these barriers. We found few meaningful differences thematically between nurses stratified by self-reported levels of comfort with provision of care to children and their families.

The most striking finding echoed across the 41 interviews was the immediacy with which hospice nurses expressed a need for pediatric-specific training and support. Nearly all nurses felt both privileged and burdened by the responsibility of caring for dying children, conveying urgency in their need for interventions to ensure provision of optimal care. These findings are a call to action for the palliative care community to collaborate in rapid implementation of educational programs and networks to systematically support hospice nurses in the field.

Nurses also stressed the importance of access to experts in pediatric hospice and palliative care. Notably, access to information was described as essential but not sufficient—community and solidarity are critical aspects of education and support. Building on nurses’ keen discernment of what matters most as they care for children in the community, we propose a training model built upon a foundation of community building. Recognizing that extensive didactic programming may not be feasible for full-time nurses, we advocate for development of a spoke-and-hub model in which pediatric academic centers partner with community hospices serving their surrounding catchment areas to bring clinicians together on a regular basis to carry out didactic learning, foster a sense of community and solidarity, help nurses network with colleagues, and reinforce access to colleagues and experts who can offer guidance in real time. Precedent exists for this type of program as seen in the Partners in Pediatric Palliative Care model^34^; the Georgia Hospice and Palliative Care Organization^35^; and the virtual, technology-mediated teleteaching Project ECHO (Extension for Community Healthcare Outcomes).^36^ Although specific processes may vary by regional collaborative, we suggest a multifaceted approach including (1) an annual retreat; (2) a monthly, 1-hour virtual meeting during which nurses present case studies from the field and ask for guidance from pediatric palliative care experts; and (3) face-to-face spin-off learning in the forms of both in-service apprenticeship with a nurse champion and one-on-one guidance from nurse mentors in patients’ homes. Crucially, to serve nurses’ multifaceted needs, the expert team designing and implementing this intervention must be interprofessional, including physicians, advanced practice health care professionals, psychologists, social workers, chaplains, and child life specialists, as well as hospice nurses with experience in pediatric care.

### Limitations

Our study had several limitations. First, findings from nurses in our tristate region are not inherently representative of hospice nurse experiences or preferences nationwide. Nonetheless, statewide data from Georgia^35^ and Nebraska^37^ corroborate our findings, suggesting that they may be representative of the experience of hospice nurses across the country. Second, we did not assess competence in the field and thus cannot know how self-reported comfort levels correlate with competence. Third, the cohort of nurses interviewed were predominantly women and racially and ethnically homogenous; the majority were White, and all were non-Hispanic. This sample is not representative of the tristate region’s overall population, although it is worth exploring in further research whether such homogeneity is representative of the hospice nurse population serving the region. Fourth, many hospice nurses had limited exposure to pediatric patients, which may constrain their ability to offer comprehensive training preferences and recommendations. Finally, our findings may underrepresent the need for pediatric-specific training and support among hospice nurses given the potential for selection bias for nurses who may have had more exposure to pediatric hospice patients.

## Conclusions

In this qualitative study, community hospice nurses expressed an urgent need and clear preferences for pediatric-specific training, awareness of barriers to training, and recommendations for circumventing these challenges. We hope that these findings will inform development and investigation of educational resources and training opportunities for nurses to enable optimal provision of palliative and hospice care to children with serious illness.

## Notes

Supplement. **eTable.** Methods for Content Analysis **eFigure.** Interview Guide
