# Supplementary material for: Community Hospice Nurses’ Perspectives on Needs, Preferences, and Challenges Related to Caring for Children With Serious Illness
Source: JAMA Netw Open. 2021 Oct 4;4(10):e2127457. doi: 10.1001/jamanetworkopen.2021.27457 (PMC8491107; doi:10.1001/jamanetworkopen.2021.27457)
Supplement: Supplement. — eTable. Methods for Content Analysis eFigure. Interview Guide [file jamanetwopen-e2127457-s001.pdf]

Supplementary Online Content

Porter AS, Zalud K, Applegarth J, et al. Community hospice nurses' perspectives on needs, preferences, and challenges related to caring for children with serious illness. *JAMA Netw Open*. 2021;4(10):e2127457. doi:10.1001/jamanetworkopen.2021.27457

eTable. Methods for Content Analysis

eFigure. Interview Guide

This supplementary material has been provided by the authors to give readers additional information about their work.

© 2021 Porter AS et al. *JAMA Network Open.*

eTable 1. Methods for Content Analysis

Phase	Methods
Codebook development	• Four researchers (E.C.K., M.G., K.Z., J.A.) reviewed transcripts, conducted memo-writing,[1] and used raw data to guide inductive development of codes and code definitions across serial meetings.[2]<br>• The codebook was finalized following deep review of sufficient raw data to reach saturation, with no new concepts emerging from transcripts.[3]
Piloting	• Three researchers (M.G., K.Z., J.A.) pilot-tested the codebook across a series of interview transcripts to identify areas of variance.<br>• Five researchers (E.C.K., M.G., K.Z., J.A.) met to reconcile variances and achieve consensus, modifying the codebook as needed to improve dependability, confirmability, and credibility of independent codes.[4]
Coding	• Following codebook finalization, independent coding was performed by three analysts with significant training and experience with content analysis (M.G., K.Z., J.A.) with weekly meetings to review any coding variances and third-party adjudication (A.P., E.K., J.B.) to reach consensus.<br>• Consistency in code segmentation also was reviewed to ensure a standardized approach (M.G., E.K.).
Synthesis & Validation	• Pattern within and between codes were examined closely, grouped into categories, and reorganized to identify patterns and themes[5] derived from hospice nurse responses (A.P., C.W., E.K.).<br>• Code frequency counts were calculated (A.P., C.W.) and presented descriptively to highlight patterns.<br>• Two nurses with community-based hospice experience (E.R., K.W.) reviewed synthesized data to provide feedback and ensure that study findings accurately captured the perspective of hospice nurses.

1. Birks M, Francis K. Memoing in qualitative research Probing data and processes. *J Res Nurs*. 2008;13(1):68-75. doi:10.1177/1744987107081254
2. Bradley EH, Curry LA, Devers KJ. Qualitative data analysis for health services research: Developing taxonomy, themes, and theory. *Health Serv Res*. 2007;42(4):1758-1772. doi:10.1111/j.1475-6773.2006.00684.x
3. Saunders B, Sim J, Kingstone T, et al. Saturation in qualitative research: exploring its conceptualization and operationalization. *Qual Quant*. 2018;52(4):1893-1907. doi:10.1007/s11135-017-0574-8
4. Korstjens I, Moser A. Series: Practical guidance to qualitative research. Part 4: Trustworthiness and publishing. *Eur J Gen Pract*. 2018;24(1):120-124. doi:10.1080/13814788.2017.1375092
5. Krippendorff K. *Content Analysis: An Introduction to Its Methodology*. Newbury Park, CA: SAGE Publications; 1980.

© 2021 Porter AS et al. *JAMA Network Open.*

eFigure 1. Interview Guide

Introduction

Thank you so much for agreeing to participate in this interview. As we previously explained, we are hoping to develop educational resources and programs to better support hospice nurses and improve pediatric palliative and hospice care in our community. We truly value your insight. As a token of our appreciation, everyone who participates in an interview will be offered a $25 Target gift card.

Please feel free to stop this interview at any point if you run out of time or simply do not want to continue the conversation; there will be no penalty for stopping. All interviews are recorded for the purposes of data analysis, but all responses are kept confidential to ensure that nothing you say during this interview can be traced back to you.

Again, we are very grateful to you for taking the time to do this. Please know that your reflections will contribute significantly to improving the experiences of patients, families, and hospice nurses in the tristate area and beyond.

Open-ended Interview Guide

1. What (if any) pediatric-specific training in palliative or hospice care have you had?
    a. In nursing school? By your past or current employer? By a third party?
    b. What did the training entail? Structure? Content?

2. Do you think you have had sufficient training to provide palliative or hospice care to pediatric patients?
    a. Why or why not?
    b. Tell me more.

3. Think back to the last time you were working with a child with palliative or hospice care needs in which you felt uncomfortable or under-prepared. Tell me about the experience.
    a. With which aspects did you feel comfortable? Uncomfortable?
    b. What skills did you use? What skills did you need but did not have?
    c. What resources did you use? What resources did you need but did not have?
        i. Written/paper?
        ii. Online or apps?
        iii. ELNEC or other courses?
        iv. People – advisors, mentors, experts, or others; apprenticeship or targeted questions and troubleshooting as needed?

4. If money, time, and logistics were not an issue, what training and/or other resources would you want to care for children with serious illness?
    a. Have you observed colleagues using resources that you wish you could access?
    b. Do you know of any trainings or workshops that you wish you could attend?

© 2021 Porter AS et al. *JAMA Network Open.*

    c. Do you know of any written, online, or app-based materials that you wish you had?

5. If you were going to design a curriculum on pediatric palliative and hospice care, what topics would be most important to you?
    a. In our recent survey, hospice nurses indicated that symptom management was one of the most important topics. Do you agree? Would you want more training on that? Why or why not?
    b. Hospice nurses also indicated that communication, psychosocial support, and bereavement support were important topics to them. Do you agree? Would you want more training on that? Why or why not?
    c. Some survey respondents stated that they would like more cultural competency training. Such a lesson might include learning about different cultural groups' ideas about illness, wellness, treatments, death, afterlife, etc. Would you be interested in including that topic in the curriculum?
        i. Why or why not?
        ii. Do you struggle to navigate cultural differences in your own work?
        iii. Do you struggle to navigate language barriers in your own work?

6. I am interested in how best to present the information on this topic. In what ways do you learn best?
    a. Lectures, seminars, or other formal didactics? In-person or virtual (e.g. webinars)? Books, papers, and other reading materials? Video? Websites, apps, or other interactive multimedia?
    b. Do you need formal evaluation after a learning experience to consolidate the lessons learned?
        i. Do you benefit from having quizzes or exams after a learning experience?
        ii. Do you benefit from having projects or papers after a learning experience?
    c. How have you seen pediatric palliative and hospice care education taught in the past? What worked well and what fell short?

7. What barriers might prevent you from accessing the types of educational resources and opportunities that you just described?
    a. Time, both amidst your many work obligations and in terms of your personal life (i.e. making time to travel to a conference)?
    b. Relatively lower importance as compared to other trainings or education that you need more (e.g. prioritizing training in adult hospice care over pediatric care)?
    c. Distance from urban centers where conferences and trainings are usually held?
    d. Personal reasons (e.g., fear of taking care of pediatric patients, lack of desire to focus on pediatric care, concern about having all pediatric patients assigned to your caseload)?
    e. Lack of institutional support from your hospice organization?

8. Have you imagined changes – small or large – that could bypass the barriers we just discussed?

© 2021 Porter AS et al. *JAMA Network Open.*

a. Have you worked in settings in which these barriers are not present? If so, what was done to prevent/overcome barriers?
b. Have you talked with colleagues who work in settings in which the barriers you face are not present? If so, what was done to prevent/overcome barriers?

9. Have you had any experiences in your ***personal life*** that have influenced the way you practice as a hospice nurse when taking care of children?
    a. What was notable or significant about those experiences?
    b. How did they influence the way you practice as a nurse?
    c. Could you use those experiences to teach someone how to practice as a nurse?

10. Have you had any experiences in your ***professional life*** that have influenced the way you practice as a hospice nurse when taking care of children?
    a. What was notable or significant about those experiences?
    b. How did they influence the way you practice as a nurse?
    c. Could you use those experiences to teach someone how to practice as a nurse?

11. As you know, we are trying to figure out how to improve training and resources to better support pediatric hospice nurses. Given then, is there anything else you want to share with us that we have not asked about during this interview?

12. What is your preferred mailing address for your $25 Target gift card?

Thank you so much for taking the time to answer these questions. We value your perspective and are very grateful to you for sharing your insights with us! Please allow 2-4 weeks for us to mail your gift card. Do not hesitate to reach out to us if you have any additional questions or comments to share either now or in the future.

© 2021 Porter AS et al. *JAMA Network Open.*
